# Usefulness of staging chest-CT in patients with operable breast cancer

**DOI:** 10.1371/journal.pone.0246563

**Published:** 2021-02-11

**Authors:** Jung Hee Hong, Jin Mo Goo, Hyeong-Gon Moon, Jung Min Chang, Jong Hyuk Lee, Chang Min Park

**Affiliations:** 1 Department of Radiology, Seoul National University College of Medicine, Seoul National University Hospital, Seoul, Korea; 2 Department of Surgery and Cancer Research Institute, Seoul National University Hospital, Seoul, Korea; Northwestern University Feinberg School of Medicine, UNITED STATES

## Abstract

**Objective:**

The aim of this study was to investigate the usefulness of staging chest-CT in terms of diagnostic yield and false-referral rate in patients with operable breast cancer.

**Materials and methods:**

This study was approved by the institutional review border. In this retrospective study, we reviewed patients who underwent staging chest-CT between January 2014 and June 2016. Reference standard was defined as a combination of pathology and radiologic tumor changes in accordance with primary tumor or metastatic lesions and stability during the 12-month follow-up period. We calculated diagnostic yield and false-referral rates stratified by pathologic stage. The important ancillary findings of staging chest-CT were also recorded.

**Results:**

A total of 1,342 patients were included in this study. Of these, four patients (0.3%; 4/1342) had true pulmonary metastasis. Diagnostic yields of stage I, II, III disease were 0.0% (0/521), 0.3% (2/693), and 1.6% (2/128), respectively. The overall false-referral rate was 4.6% (62/1342); false-referral rates of stage I, II, and III disease were 5.0% (26/521), 3.8% (26/693), and 7.8% (10/128), respectively. No occult thoracic metastasis occurred within 12 months of staging chest-CT. Nineteen patients showed significant ancillary findings besides lung metastasis, including primary lung cancer (n = 9). The overall diagnostic yield of ancillary findings was 1.7% (23 of 1342).

**Conclusions:**

The incidence of pulmonary metastasis was near zero for pathologic stages I/II and slightly higher (although still low; 1.6%). for stage III. Considering its low diagnostic yield and substantial false-referral rates, staging chest-CT might not be useful in patients with operable breast cancer.

## Introduction

Breast cancer is the most common cause of cancer deaths in women worldwide; most cases are detected at an early stage when no distant metastasis has occurred [1, 2]. Patients with early breast cancer have a chance to be treated with curative surgery or locoregional therapy; the 5-year survival rate (85–99%) in these patients is much higher than those with distant metastasis (25%). Therefore, accurate detection of distant metastasis will help to implement the treatment plan and prognostication; however, only about 4–6% of patients with breast cancer have evidence of distant metastasis at initial diagnosis [2–6].

After bone, the lung is the second most common distant metastatic site in patients with breast cancer, followed by the brain and liver [6]. However, the prevalence of radiologically-evident distant lung metastasis in early stage breast cancer is low, approximately 0.2% and 1.2% for stage I and II disease, respectively [3, 7–11]. Most of the existing guidelines, including the National Comprehensive Cancer Network (NCCN), European Society for Medical Oncology (ESMO), and American Society of Clinical Oncology (ASCO), recommend staging chest-CT only for advanced disease [12–14]. Despite these recommendations, many patients diagnosed with early stage breast cancer often undergo chest-CT as a part of initial staging evaluation [11, 15, 16]. The majority of physicians are aware of the published guidelines, but they do not change their staging imaging practices; moreover, the majority of patients also prefer to undergo chest-CT [17, 18].

To reduce the discrepancy between the guidelines and routine clinical practice, it is necessary to provide evidence for the benefit of the chest-CT according to the stages of breast cancer. Therefore, we investigated the usefulness of chest-CT as staging work-up in terms of diagnostic yield and false-referral rates among the patients who underwent chest-CT scan.

## Materials and methods

This retrospective study was approved by the institutional review board of Seoul National University Hospital; the requirement for patients’ informed consent was waived. All data were fully anonymized before we assessed them.

### Study design and patients

**Fig 1:**
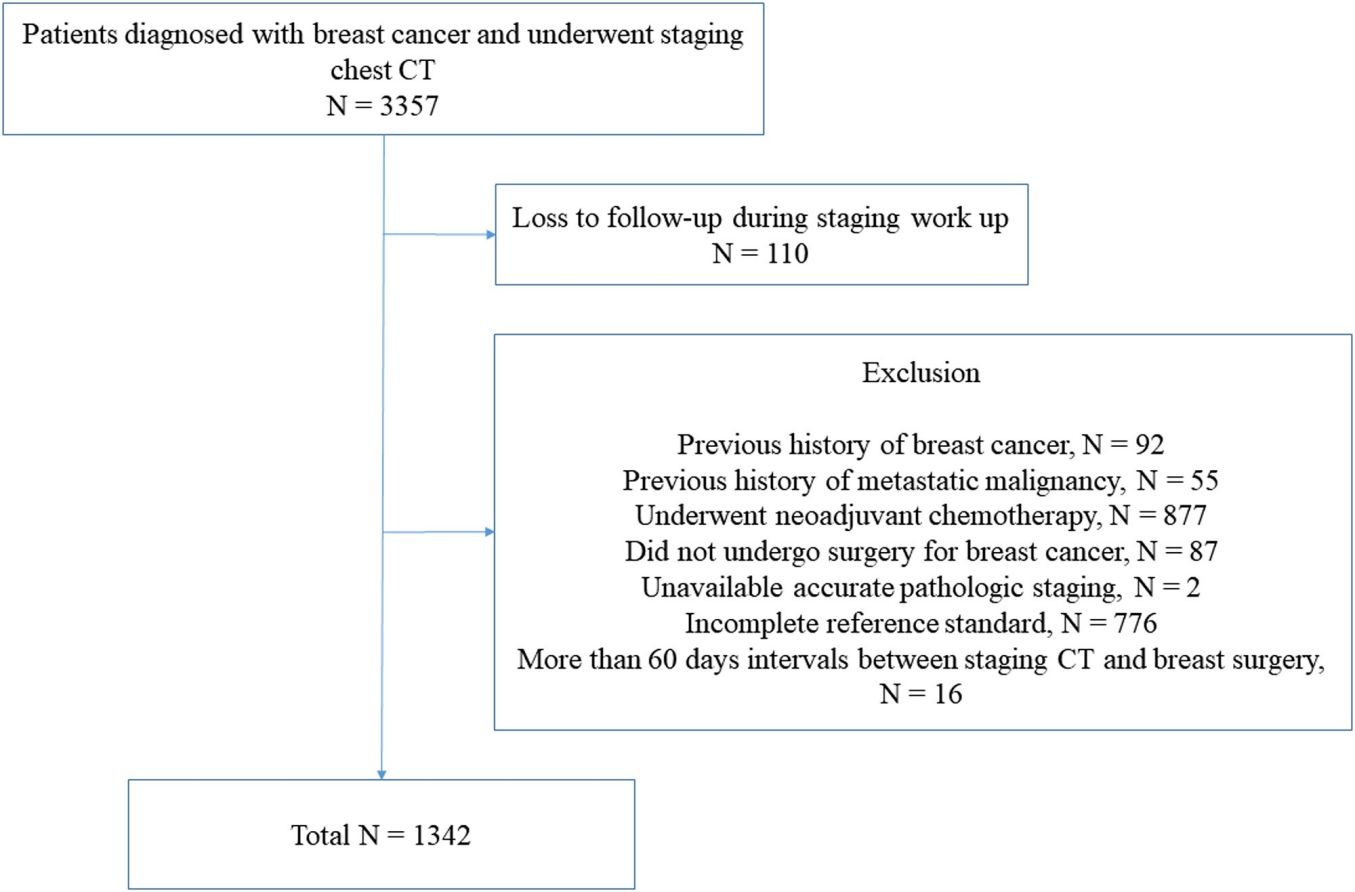
Flow diagram showing the patient selection process with inclusion and exclusion criteria.

A retrospective review of patients with operable breast cancer was performed at a single tertiary hospital. Eligibility criteria were as follows: 1) patients were diagnosed with primary breast cancer from January 2014 to June 2016 and 2) patients underwent chest-CT for staging work-ups with or without other diagnostic imaging studies. We identified of 3357 consecutive patients during the study period. Among them, we excluded the patients who 1) were lost to follow-up during the staging work-up (n = 110), 2) had a previous history of breast cancer (n = 92), c) had a previous history of malignancy, other than breast cancer with a potential to metastasize (n = 55), and 4) received neoadjuvant systemic therapy (n = 877). The patients who did not undergo surgery for breast cancer (n = 87) who already diagnosed as non-operable advanced breast cancer by their initial work-ups (physical exam, symptoms, mammography and ultrasound) and subsequent further diagnostic work-ups were also excluded. Patients who did not have an available pathologic stage (n = 2) were excluded for accurate stratification of tumor stage. The patients who did not fulfill the criteria for determining the thoracic metastasis described below (n = 776) were excluded. In addition, 16 patients who had >60 days interval between staging CT and breast surgery were also excluded (Fig 1).

All patients underwent chest-CT with ≥16-channel multi-detector CT scanners, one of the seven different CT scanners at our institute (Sensation 16, SOMATOM Definition, Siemens Medical Solutions, Forchheim, Germany; Brilliance-64, Ingenuity, Philips Medical System, Best Netherlands; Aquilion One, Toshiba, Japan; Discovery CT750HD, LightSpeed Ultra, GE Medical Systems, Waukesha, Wis). In the majority of patients, CT was performed using a fixed tube voltage of 120 kVp, with automatic exposure control, an image slice thickness of 1–5 mm, and with intravenous contrast material administration.

### Clinical and radiological analysis

**Fig 2:**
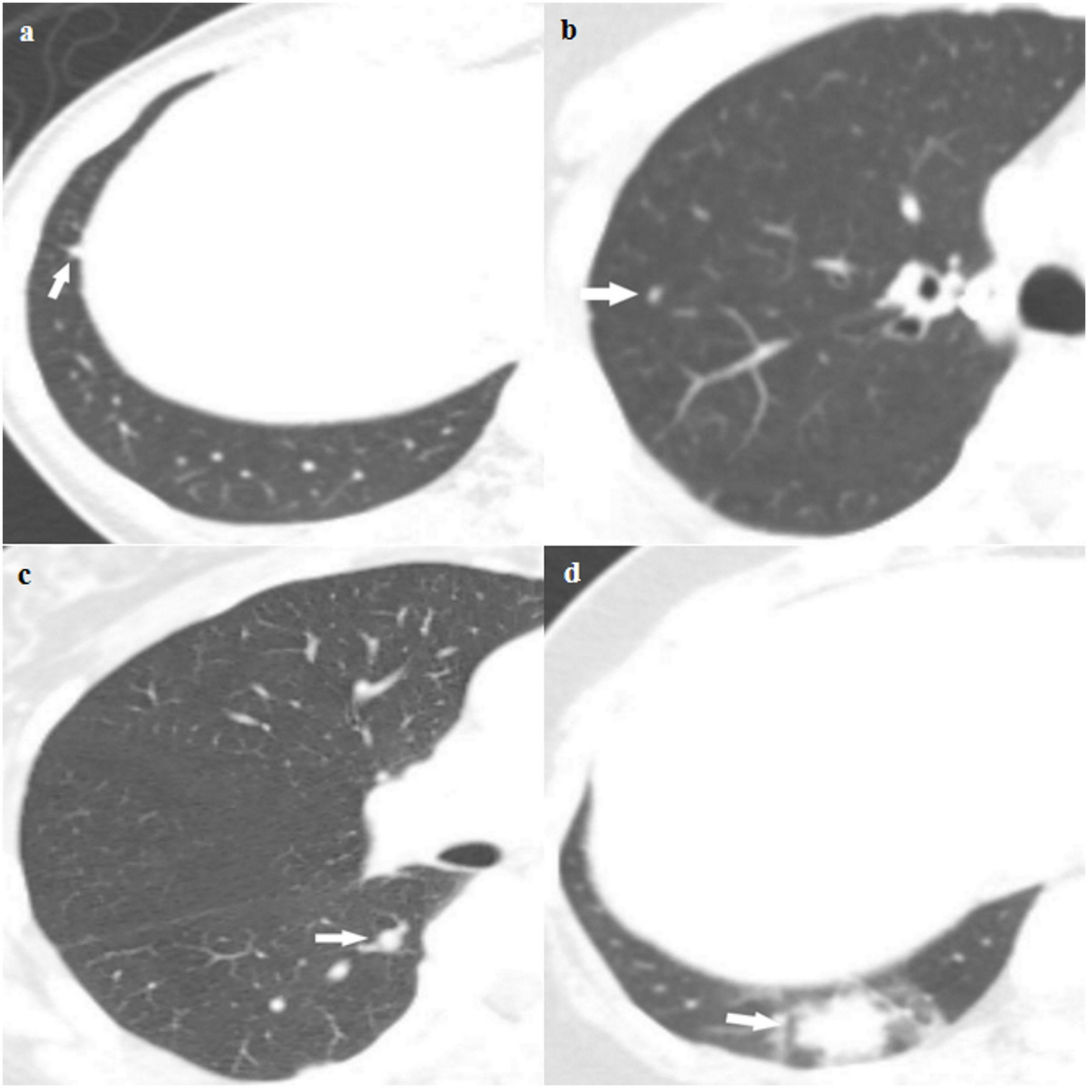
Representative examples of the four-point scaling likelihood scores for a (a) very low level of suspicion (including those without pulmonary nodules); (b) low level of suspicion; (c) indeterminate level of suspicion; and (d) definite metastasis. (a) Score 1. Axial staging chest-CT scan of a 53-year-old female with pathologic stage I breast cancer showing a 4-mm sized polygonal shaped nodule with a thin tag extending to the pleura in the right lower lobe, which probably represents an intrapulmonary lymph node. (b) Score 2. Axial staging chest-CT scan of a 72-year-old female with pathologic stage II breast cancer showing a 3-mm sized solid nodule in the right upper lobe. The likelihood score for presence of metastasis was score 2. (c) Score 3. Axial staging chest-CT scan of a 59-year-old female with pathologic stage I breast cancer showing a 6-mm sized solid nodule in the right lower lobe, which was classified as score 3. (d) Score 4. Axial staging chest-CT scan of a 39-year-old female with pathologic stage II breast cancer showing a 22-mm-sized lobulating nodule in the right lower lobe with ground-glass opacities. This nodule subsequently disappeared on follow-up chest-CT and was finally diagnosed as a benign inflammatory nodule.

One radiologist (J.H.H. with 6 year experience in chest imaging), who was blinded to the reference standard results, evaluated staging chest-CT scans for nodule analysis by taking into consideration patient’s clinical information and radiology reports of chest-CT scans. The likelihood of the presence of lung metastasis was recorded using a four-point scaling score: 1, very low level of suspicion (including those without pulmonary nodule); 2, low level of suspicion; 3, indeterminate level of suspicion; and 4, definite metastasis (Fig 2). Sub-solid nodules rarely represent pulmonary metastasis; therefore, only solid nodules were assigned a score of 3 or 4 [19, 20]. We considered the two lower scores (score 1 and 2) as a negative result and the two higher scores (score 3 and 4) as a positive result [21].

The size of the nodule with the highest likelihood score was manually measured as the longest axial diameter. The number of lung nodules was checked and categorized as “group a” with 1–5 nodules, “group b” with 6–10 nodules, and “group c” with >10 nodules. We also checked the number of lung nodules with respect to whether the nodules were solitary (n = 1) or multiple (n>1). Additionally, other important ancillary findings of chest-CT scans were recorded.

A research assistant searched the electronic medical records and collected patient information such as age, histologic tumor grade, estrogen receptor (ER), progesterone receptor (PR), human epidermal growth factor receptor 2 (HER2) status, and pathologic data, including pathologic T and N categories. The pathological anatomical staging was allocated according to the eighth edition of the TNM staging system of the American Joint Committee on Cancer (AJCC) [22].

Pulmonary nodules were classified as true metastasis if the nodules were: 1) pathologically confirmed as metastasis, 2) increased in size on follow-up CT scan, 3) decreased in size after chemotherapy in accordance with primary tumor or metastatic lesions, and 4) showed hypermetabolism (the maximum standardized uptake value more than 2.5) on 18F-fluorodeoxyglucose (FDG) positron emission tomography (PET)/CT with suspected of metastasis by nuclear medicine doctor, and were clinically considered metastasis [21]. Pulmonary lesions were classified as true negative for lung metastasis if the following criteria were satisfied: 1) nodules were pathologically confirmed as negative for metastasis, 2) nodules decreased in size without treatment, and 3) nodules remained stable for ≥12 months as determined by follow-up CT [21]. Patients who failed to meet any of the above criteria were considered indeterminate and excluded (n = 776).

### Statistical analysis

To determine the usefulness of staging chest-CT, diagnostic yield and false-referral rates were evaluated, which indicated true positive or false positive results. Diagnostic yield was calculated as the number of patients with true positive results for lung metastasis divided by the number of eligible patients who underwent staging chest-CT. False-referral rate was calculated as the number of patients with false positive results for lung metastasis divided by the number of eligible patients who underwent staging chest-CT [21, 23]. Incidentally detected primary lung cancer was considered as an important ancillary finding and the data were recorded separately from lung metastasis. Occult metastasis was diagnosed using staging chest-CT and was defined as thoracic metastasis occurring within 12-months of staging CT.

A Mann-Whitney U-test was applied to assess continuous variables. The Fisher’s exact test was used to assess categorical variables. *P*<.05 was considered statistically significant. Analyses were performed using the SPSS software (IBM Corp. Released 2015. IBM SPSS Statistics for Windows, Version 23.0. Armonk, NY: IBM Corp).

## Results

Clinical characteristics of 1,342 patients are summarized in Table 1. Mean age of the patients was 50.9 years (range, 23–83 years; 4 men and 1,338 women). The number of patients with pathologic stage IA, IB, IIA, IIB, IIIA, IIIB, and IIIC disease were 516 (38.5%), 5 (0.4%), 483 (36.0%), 210 (15.7%), 87 (6.5%), 3 (0.2%), and 38 (2.8%), respectively.

**Table 1:** Clinical characteristics of study population.

Pathologic stage	Total		Stage IA		Stage IB		Stage IIA		Stage IIB		Stage IIIA		Stage IIIB		Stage IIIC	
**Number of patients (%)**	1342		516 (38.5)		5 (0.4)		483 (36.0)		210 (15.7)		87 (6.5)		3 (0.2)		38 (2.8)	
Age at diagnosis*	50.9 ± 10.6		50.5 ± 10.2		64.2 ± 11.1		50.7 ± 10.4		50.3 ± 10.2		51.7 ± 11.1		50.0 ± 19.2		56.6 ± 14.6	
**Histologic tumor grade**																
Grade I	109	8.1	63	12.2	0	0.0	29	6.0	10	4.8	6	6.9	0	0.0	1	2.6
Grade II	675	50.4	250	48.5	3	60.0	233	48.2	117	55.7	49	56.3	2	66.7	21	55.3
Grade III	555	41.4	202	39.2	2	40.0	221	45.8	83	39.5	32	36.8	1	33.3	16	42.1
Unknown	1	0.1	1	0.2												
**ER status**																
Positive	969	72.3	350	67.8	2	40.0	335	69.4	174	82.9	76	87.4	2	66.7	31	81.6
Negative	371	27.7	166	32.2	3	60.0	148	30.6	36	17.1	11	12.6	1	33.3	7	18.4
Unknown																
**PR status**																
Positive	794	59.3	284	55.0	2	40.0	267	55.3	156	74.3	62	71.3	2	66.7	21	55.3
Negative	546	40.7	232	45.0	3	60.0	216	44.7	54	25.7	25	28.7	1	33.3	17	44.7
Unknown																
**HER2 status**																
Positive	282	20.0	122	23.6	1	20.0	100	20.7	28	86.7	17	19.5	0	0.0	14	36.8
Negative	1051	78.4	389	75.4	3	60.0	383	79.3	182	13.3	69	79.3	3	100.0	24	63.2
Unknown	7	0.5	5	1.0	1	20.0					1	1.1				

Note—ER = estrogen receptor; PR = progesterone receptor; HER2 = human epidermal growth factor receptor 2.

**Fig 3:**
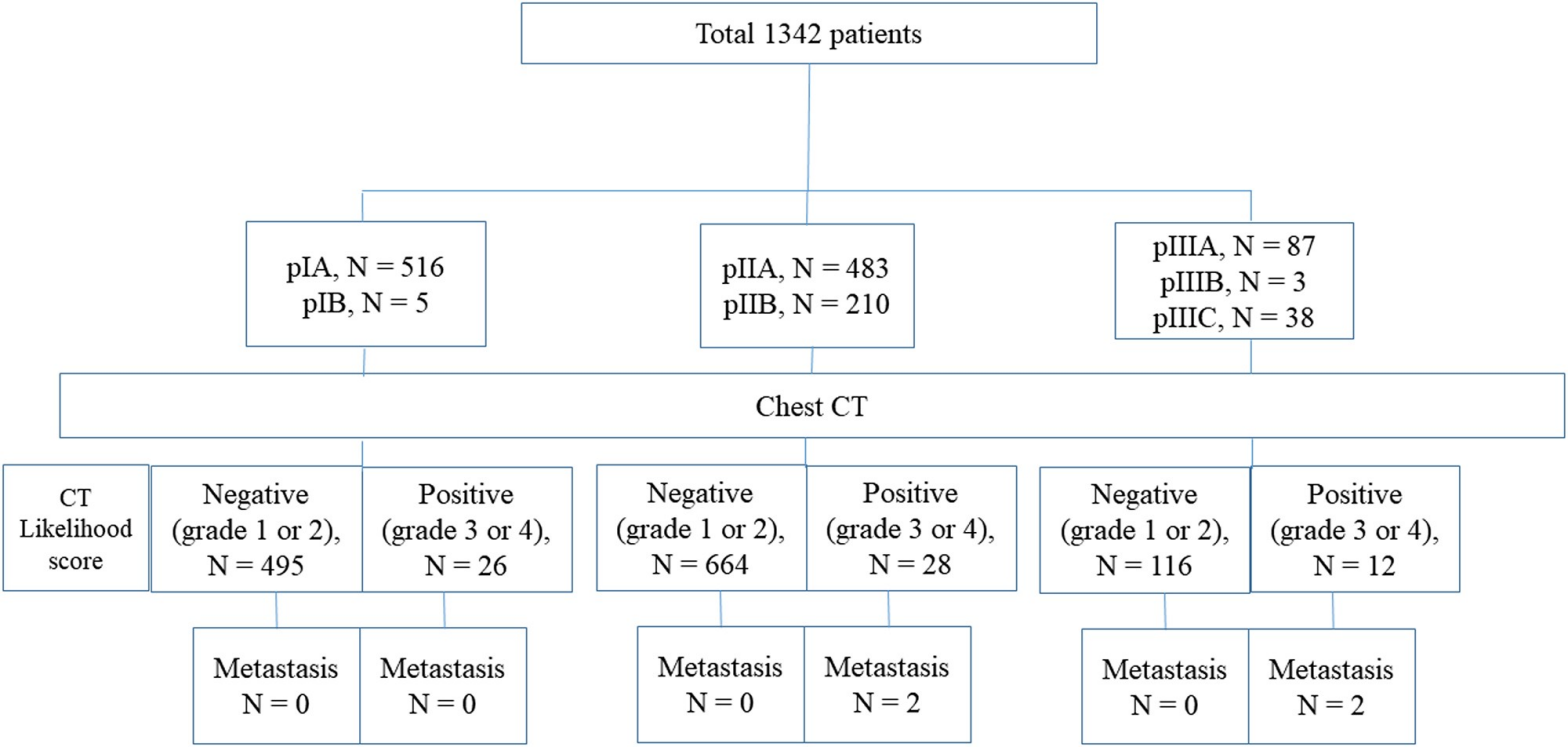
Patient flow diagram stratified according to pathologic stage and presence of metastatic lung nodule.

**Fig 4:**
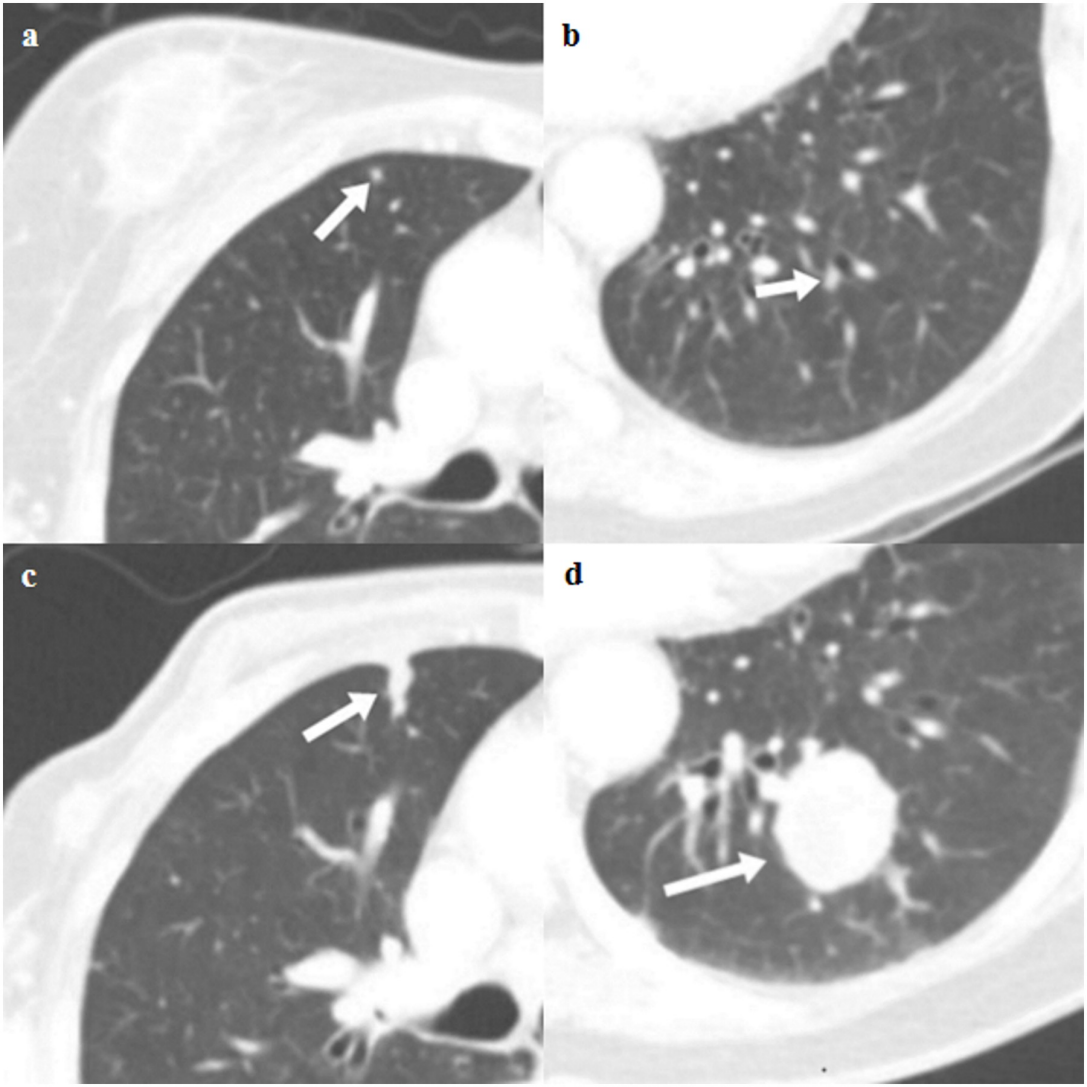
Axial CT images of true lung metastases in a 78-year-old female with pathologic stage III breast cancer. (a and b) Staging chest CT images show two small nodules (arrow) in the right middle lobe and left lower lobe (indeterminate). (c and d) Follow-up chest CT images show growth of two nodules (arrow), which are thought to be pulmonary metastasis.

**Fig 5:**
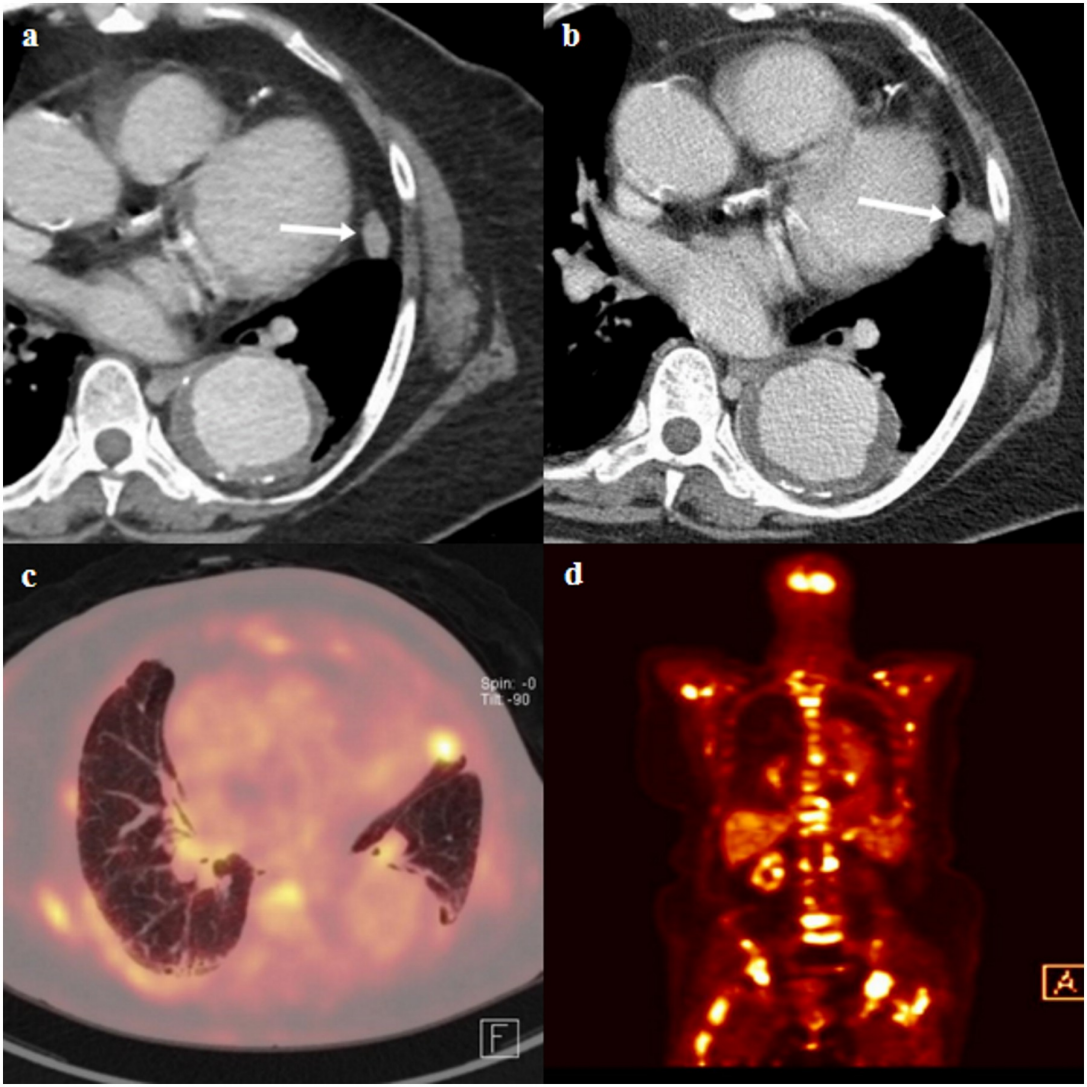
CT and fluorine 18 fluorodeoxygluocose (FDG) positron emission tomography (PET)/CT images of a true lung metastasis of a 71-year-old female with pathologic stage III breast cancer. (a) Staging chest-CT image shows an enhancing indeterminate nodule (arrow) in left upper lobe lingular segment. In addition, descending thoracic aortic aneurysm was incidentally detected. (b) A follow-up chest-CT image shows an interval growth of nodule (arrow). (c and d) Axial and coronal FDG PET/CT scans show FDG uptake in a growing nodule in the left upper lobe lingular segment (SUV_max_, 7.7) and multiple axial bony thorax (SUV_max_, 12.1). The bony lesion of the left ilium (not shown) was pathologically confirmed as metastasis after CT-guided biopsy. The nodule in the left upper lobe lingular segment was subsequently regarded as pulmonary metastasis.

Of the 1,342 patients, 66 patients had positive results on staging chest-CT scan (4.9%). Overall, four patients (0.3%) were considered as “true positive” by our criteria; 3 of 4 were diagnosed with an increased size of nodules on follow-up CT scan with increased metabolism on PET-CT and 1 of 4 was decreased in size after chemotherapy in accordance with primary tumor or metastatic lesion (Figs 3–5). Seven patients (0.5%) who had false positive results underwent an invasive procedure; one patient underwent pulmonary wedge resection, but it was diagnosed as chronic granulomatous inflammation; two patients underwent percutaneous lung biopsy and were diagnosed with chronic granulomatous inflammation and sclerosing pneumocytoma, respectively; and the remaining four patients were diagnosed with primary lung cancer. There was no detected mediastinal lymph node metastases or bone metastatic lesions.

The overall diagnostic yield of staging chest-CT scan was 0.3% (4/1342; 95% confidence interval [CI]: 0.0%, 0.8%). Analyzed according to the pathologic stage, the diagnostic yield of stage I, II, III was 0.0% (0/521; 95% CI: 0.0%, 0.0%), 0.3% (2/693; 95% CI: 0.0%, 1.0%), and 1.6% (2/128; 95% CI: 0.2%, 5.5%), respectively. Overall false-referral rate was 4.6% (62/1342; 95% CI: 3.6%, 5.9%); analyzed according to the pathologic stage, the false-referral rate of stage I, II, and III were 5.0% (26/521; 95% CI: 3.3%, 7.2%), 3.8% (26/693; 95% CI, 2.5%, 5.5%), and 7.8% (10/128; 95% CI: 3.8%, 14.0%), respectively. There was no occult thoracic metastasis which occurred within 12-months after staging chest-CT scan. The sensitivity, specificity, positive predictive values, and negative predictive values of staging chest-CT scan were 100%, 95.4%, 6.0%, and 100%, respectively.

Age at diagnosis (*P* = .003) and pathologic stage (*P* = .015) were significantly different between patients with pulmonary metastasis (n = 4) and those without metastasis (n = 1,338). Radiologic findings, including nodule size, nodule count, histologic tumor grade and ER, PR, and HER2 status were not significantly different between the two groups (Table 2).

**Table 2:** Comparison of patients with pulmonary metastasis versus without pulmonary metastasis.

	Patient with pulmonary metastasis	Patient without pulmonary metastasis	*p-*value
**Total**	4	1338	
Age at diagnosis*	66.5 ± 14.1	50.8 ± 10.5	0.003
**Pathologic stage**			0.015
I	0	521	
II	2	691	
III	2	126	
**Histologic tumor grade**			0.059
Grade I	0	109	
Grade II	0	675	
Grade III	4	553	
Unknown		1	
**ER status**			0.662
Positive	2	968	
Negative	2	370	
Unknown			
**PR status**			
Positive	1	793	0.377
Negative	3	545	
Unknown			
**HER2 status**			0.577
Positive	0	282	
Negative	4	1049	
Unknown		7	
**Presence of lung nodule**	4	467	
Positive results on CT	4	62	
**Nodule size**†(mm)	4.5 (IQR, 2.3–7.5)	3.0 (IQR, 3.0–4.0)	0.450
**Nodule count**††			0.707
Group a	4	398	
Group b	0	41	
Group c	0	28	
**Nodule count**††			0.343
Single	0	165	
Multiple	4	302	

Note—ER = estrogen receptor; PR = progesterone receptor; HER2 = human epidermal growth factor receptor 2, IQR = interquartile range.

*Data are expressed as arithmetic mean ± standard deviation.

^†^Data are expressed as the median and interquartile range (IQR).

^††^Total number of patients with nodules were 467 patients and the remaining 871 patients did not have any nodules on staging chest CT.

Among 1,342 patients, 19 patients had significant ancillary findings besides lung metastasis (Table 3). Primary lung cancer (n = 9), vascular anomaly (n = 6), hepatocellular carcinoma (n = 2), and interstitial lung disease (n = 3) were incidentally detected. Among the nine nodules diagnosed as primary lung cancer, four were manifested as solid nodule, four were sub-solid nodules, and one was manifested as asymmetric bronchial wall thickening. Eight patients were never-smokers and one was current smoker with a smoking history of 10 pack-year. The overall diagnostic yield considering ancillary findings in addition to pulmonary metastasis was 1.7% (23/1,342; 95% CI: 1.1%, 2.6%). When stratified by pathologic stage, diagnostic yield considering important ancillary findings in addition to true positive pulmonary metastasis in pathologic stages I, II, and III were 2.1% (11/521; 95% CI: 1.1%, 3.8%), 1.3% (9/693; 95% CI: 0.6%, 2.5%), and 2.3% (3/128; 95% CI: 0.5%, 6.7%), respectively.

**Table 3:** Significant ancillary findings on chest CT.

Significant ancillary findings	
Primary lung cancer	9
Vascular anomaly	6
Hepatocellular carcinoma	2
Interstitial lung disease	3

Note—One patient had both lung cancer and vascular anomaly.

## Discussion

The incidence of distant metastasis in patients with early breast cancer has been reported to be low [3, 7, 8, 11]. Furthermore, there was no added survival value of additional staging studies [24]. Therefore, most guidelines do not recommend routine staging work-up using chest-CT for early breast cancer [12–14, 25]. However, imaging work-up for distant metastasis has frequently been performed in clinical practice, especially in patients with higher tumor stage, younger age, and unfavorable tumor markers [10, 11, 15, 16, 26, 27].

We found that the incidence of pulmonary metastasis was near zero in patients with pathologic stage I/II and slightly higher, although still low (1.6%), in those with stage III breast cancer. Even when the important ancillary findings were considered along with lung metastasis, the diagnostic yield of staging chest-CT scan was low (overall, 1.7%; 2.1%, 1.3%, and 2.3%, for stage I, II and III, respectively). Therefore, this low diagnostic yield of staging chest-CT in operable breast cancer support the NCCN guidelines that do not recommend chest imaging for stages I, II, or IIIA (T3N1) breast cancer without symptoms or signs suggesting metastatic diseases [14]. Furthermore, our results suggest that staging chest-CT might not be beneficial for patients with operable breast cancer in pathologic stage IIIB and IIIC and could be omitted for staging work-up.

We found that patients who had pulmonary metastases were older. Interestingly, there was no significant difference in hormone markers including ER, PR, and HER2 amplification between the two groups. Although the number of patients with pulmonary metastases was very small in our study, this result was consistent with those of previous studies that showed a positive relationship between age and lung metastasis; other viscera and bone metastasis showed an inverse relationship [11, 28, 29]. In addition, our study highlights that younger age and hormonal markers, which were associated with aggressive tumor characteristics, were not a relevant factors in terms of pulmonary metastasis [10, 11, 15, 26, 27]. This also supports the NCCN guidelines that do not address hormonal markers and younger age.

As the advances in CT technology led to reduction in radiation dose and improvement of diagnostic accuracy, the use of chest-CT is gradually increasing [30]. In contrast to the established guidelines for screening- or incidentally-detected pulmonary nodules, there has been no consensus on the management of indeterminate pulmonary nodules in patients with underlying malignancies such as breast cancer [31, 32]. Guidelines for incidentally- or screening-detected pulmonary nodules consider nodule characteristics such as size, number, and nodule type as important imaging markers for determining management plan. However, our result showed that nodule size and respective groups of nodule number were not associated with pulmonary metastasis. Interestingly, a previous study reported that in patients with breast cancer, either multiple nodules or nodule size >10 mm increase the likelihood of metastasis over single or <10 mm-sized nodules [33]. Although there was no solitary nodule that was confirmed as pulmonary metastasis in this study, the difference in the likelihood of pulmonary metastasis among patients with solitary versus multiple nodules was statistically insignificant (*P* = .343). In addition, none of the patients with nodules >10 mm had pulmonary metastasis, indicating no significant difference in the proportion of pulmonary metastasis in these two size categories (≥10 mm vs. <10 mm: 0/4 vs. 11/1338; *P* = 1.000). The difference between this and the previous report may arise from the different characteristics of the study population and the small number patients with metastasis in our study. Unlike the previous study, we excluded patients who underwent pre-operative treatment. Further study will be required to assess the association between size, number of nodules, and the likelihood of malignancy [33].

The strength of our study is that we estimated the false-referral rate in addition to diagnostic yield of staging chest-CT to evaluate its potential disadvantage. Additionally, as we assessed important ancillary findings other than metastasis, we could estimate the diagnostic accuracy of clinically meaningful CT findings, in addition to pulmonary metastasis. Finally, we defined the reference standard based on the pre-existing criteria, which were carefully determined by a multidisciplinary team discussion from a previous study [21].

This study had several limitations. First, this was a retrospective study conducted in a single institution. Second, the incidence of pulmonary metastasis was very low, although we included a large number of patients. Third, we did not include patients who underwent neoadjuvant chemotherapy. The optimal use of neoadjuvant chemotherapy remains unclear. In this context, some patients with neoadjuvant chemotherapy might or might not need staging chest-CT [34, 35]. Thus, exclusion of such patients with neoadjuvant chemotherapy might lead to a selection bias. Fourth, the positive CT findings were determined not based on a specific CT finding such as size or nodule morphology but on the four-point likelihood score by thoracic radiologist. However, we thought that this approach would reflect the real clinical practice better. Fifth, we excluded 776 patients with incomplete reference standard. All 776 excluded patients did not have follow-up chest CT or PET-CT for at least 12 months after staging chest-CT. Consequently, it was not possible to determine with certainty whether the detected nodule was “true positive or not”. Sixth, we classified “true positive” and “true negative” nodules according to criteria defined by results of chest-CT, PET-CT findings in addition to the surgical results. In fact, the nodule can only be concluded as “true negative” or “true positive” if it has been surgically resected. Nevertheless, surgical resection of small pulmonary nodules for pathologic diagnosis in breast cancer rarely happens in clinical practice; most of them were diagnosed with follow-up chest-CT, FDG PET-CT, or percutaneous or endobronchial biopsy.

In conclusion, the staging chest CT might not be useful in patients with operable breast cancer considering its low diagnostic yield and substantial number of false-referral rates.

## Supporting information

S1 Data (XLSX)
